# Free *N*-glycans occurring in plant extracellular fluid or cytosol interact with an auxin, indole-3-acetic acid: Putative biofunction of free *N*-glycans in plants

**DOI:** 10.5511/plantbiotechnology.25.0116a

**Published:** 2025-03-25

**Authors:** Yumeka Iguchi, Akari Horiguchi, Miran Nakano, Megumi Maeda, Akihiro Ishiwata, Yukishige Ito, Yoshinobu Kimura

**Affiliations:** 1 Department of Biofunctional Chemistry, Graduate School of Environmental and Life Science, Okayama University, 1-1-1 Tsushima-Naka, Kita-ku, Okayama 700-8530, Japan; 2 Faculty of Agriculture, Division of Agricultural Science, Okayama University, 1-1-1 Tsushima-Naka, Kita-ku, Okayama 700-8530, Japan; 3 RIKEN, Cluster for Pioneering Research, 2-1 Hirosawa, Wako, Saitama 351-0198, Japan; 4 Graduate School of Science, Osaka University, 1-1 Machikaneyama-cho, Toyonaka, Osaka 560-0043 Japan;; 5 Faculty of Food Culture, Kurashiki Sakuyo University, 3515 Nagao, Tamashima, Kurashiki, Okayama 710-0292 Japan

**Keywords:** auxin, free *N*-glycan, indole-3-acetic acid, plant *N*-glycan

## Abstract

Two types of free *N*-glycans (FNGs), high mannose type (HMT) and plant complex type (PTC), occur ubiquitously in plants, the former mainly in the cytoplasm and the latter in the vacuole or extracellular fluid. It has been hypothesized that these plant FNGs have auxin-like activity that promotes fruit ripening based on the experimental results of adding FNGs to plant tissues; however, the postulated biological functions have not been proven at this time. In this study, using fluorescence analysis in vitro, we found that Man_3_Fuc_1_Xyl_1_GlcNAc_2_ (a PCT-FNG) occurring in plant extracellular fluids, significantly decreased the fluorescence intensity of IAA in a concentration-dependent manner at acidic (extracellular fluid) and neutral pH (cytosol), suggesting that this FNG interacts with IAA. These results suggest a possibility that the interaction of PCT-FNG and IAA may reduce the hydrophobicity of IAA in acidic environments and support the movement of IAA in plant extracellular fluids. The Interactions with IAA bearing the indole ring, appear to be unique to free *N*-glycans, since they were not for other oligosaccharides such as sucrose, lactose, or chitooligosaccharides. Some other PCT-FNGs and HMT-FNGs found in plants have also been confirmed to interact with IAA, suggesting that the common trimannosyl core structure of FNGs may be a prerequisite for such interactions.

Free *N*-glycans (FNGs) are ubiquitous in plants and animals. It has been hypothesized that these plant FNGs have auxin-like activities that promote or delay fruit ripening (Priem and Gross 1992; Yunovitz and Gross 1994). A possible interaction between IAA and high-mannose type (HMT)-FNG (Man_5_GlcNAc_1_) was once speculated from the observation that Man_5_GlcNAc_1_ administration to pericarp disc of tomato fruit inhibited the IAA-induced fruit ripening and such inhibition was suppressed by tomato lectin (a chitooligosaccharide-specific lectin but not Man-specific one), but the physiological function of plant *N*-glycans remains unknown. We have previously reported that high-mannose type FNGs (HMT-FNGs) induce protein folding and inhibit amyloid aggregation in denatured proteins (Katsube et al. 2017; Kosaka et al. 2022; Tanaka et al. 2015). We also found that plant complex-type free *N*-glycans (PCT-FNGs) occur mainly in the extracellular fluid (Maeda et al. 2010; Tsujimori et al. 2019); however, whether these PCT-FNGs perform biological activities remains unclear.

During analysis of the chaperone-like activity of several FNG types, we found that PCT-FNGs, which have neither chaperone-like nor amyloid aggregation inhibitory activity, interact with tryptophan residues in denatured proteins (Katsube et al. 2017; Tanaka et al. 2015). This suggests that the PCT-FNGs also interact with compounds containing indole rings. In this study, we analyzed whether PCT-FNGs interact with indole-3-acetic acid (IAA), a plant hormone involved in plant growth and fruit ripening, which is present in the extracellular fluid during cell–cell polar transfer.

A typical plant complex-type *N*-glycan, M3FX, was prepared via hydrazinolysis of glycopeptides derived from the storage glycoproteins of *Ginkgo biloba* seeds (Maeda et al. 2013). GN2-HMT-FNGs (with Man_9_GlcNAc_2_ as the major component) were prepared from a royal jelly glycoprotein (apisin) by hydrazinolysis and Con A-Sepharose affinity chromatography (Kimura et al. 1995, 2000). GN1-HMT-FNGs were prepared from GN2-HMT-FNGs via Endo-H digestion. Briefly, partially purified GN2-HMT-FNGs (approximately 360 mg) from royal jelly glycoproteins (Kimura et al. 1998) were incubated with Endo-H (10,000 units; New England BioLabs, Ipswich, MA, USA) in 20 ml of 50 mM MES buffer (pH 5.0), containing 5 mM EDTA, at 37°C for 48 h. The resulting GN1-HMT-FNGs were subjected to gel filtration (Sephadex G-25 column) to remove the enzyme and GlcNAc residue. As a result, 37.6 mg of GN1-HMT-FNGs (Man_9_GlcNAc_1_ as a major component, more than 90%) was obtained. The structures of these HMT-FNGs were confirmed by two-dimensional high-performance liquid chromatography and mass spectrometry analysis, as described in our previous reports (Kimura et al. 1995, Kimura and Matsuo 2000). GNM3FX, GNM3X, and GNM3 were synthesized and supplied by RIKEN (Wako, Japan; Nakano et al. 2007); chitooligosaccharides (*N,N*′-diacetylchitobiose (GlcNAc_2_) and GlcNAc_4_) were generously provided by Dr. Takeshi Yamagami (Kyushu University, Fukuoka, Japan); and IAA was obtained from Nacalai Tesque (Kyoto, Japan). The interactions of monosaccharides and oligosaccharides with Trp residues intrinsic to proteins, such as various glycosidases and lectins, have conventionally been analyzed by changes in the fluorescence intensity of Trp residues (Abe et al. 2013; Hatakeyama et al. 2011; Kimura et al. 1998). In this study, therefore, the interactions between sugars and IAA were analyzed using fluorescence spectrophotometry (JASCO, Tokyo, Japan). IAA was dissolved in 100% acetonitrile and diluted with 25 mM acetic acid buffer (pH 5.6) or phosphate-buffered saline (pH 7.2) to produce 20 µM IAA. The IAA solution (3 µl) was mixed with 30 µl of various concentrations (0–10 mM) of the oligosaccharide samples. The final IAA concentration was 2 µM. IAA was excited at 280 nm and the emission spectrum was recorded from 300 to 420 nm at a constant temperature of 30°C.

First, we confirmed whether free tryptophan (a precursor of IAA) interacts with a free *N*-glycan (M3FX) and GlcNAc_2_ (reducing terminal unit of *N*-glycans). Of note, as shown in Figure 1, an interaction was also observed between free tryptophan and M3FX, but not between free tryptophan and GlcNAc_2_, suggesting that the indole ring interacts with characteristic glycan structures (for example, the common core structure of *N*-glycan).

**Figure 1:**
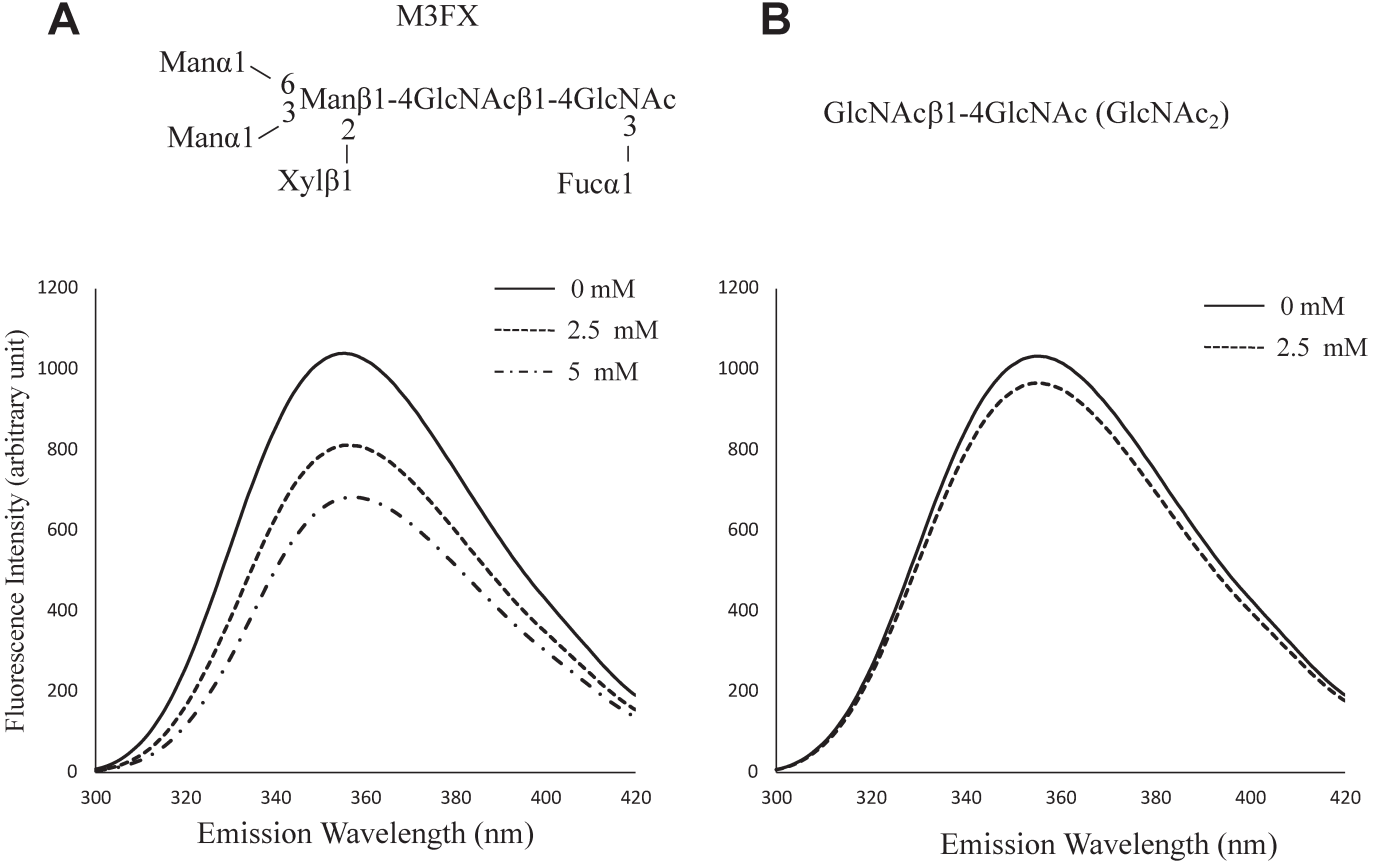
Figure 1. Interaction of M3FX with free tryptophan. A, Changes in the fluorescence spectra of indole-3-acetic acid (IAA) upon addition of M3FX (2.5, 5.0 mM) at pH 5.6. B, Changes in the fluorescence spectra of IAA upon the addition of GlcNAc_2_

We then analyzed the specific interactions between IAA and a typical PCT-FNG (M3FX) occurring ubiquitously in plants. As shown in Figure 2, the addition of M3FX caused significant quenching of IAA fluorescence in a dose-dependent manner at both neutral (cytosol, Figure 1A) and acidic pH (in extracellular fluid, Figure 1B), indicating that M3FX specifically interacted with or bound to IAA. A remarkable quenching effect of M3FX on IAA fluorescence and a red shift in the maximum fluorescence wavelength, in a glycan-concentration-dependent manner, were observed at an acidic pH. Interestingly, the decrease in fluorescence intensity of the indole ring at the same concentration of M3FX (e.g. 5 mM) was remarkably larger for IAA than for Trp, suggesting that M3FX may interact more strongly with IAA than Trp.

**Figure 2:**
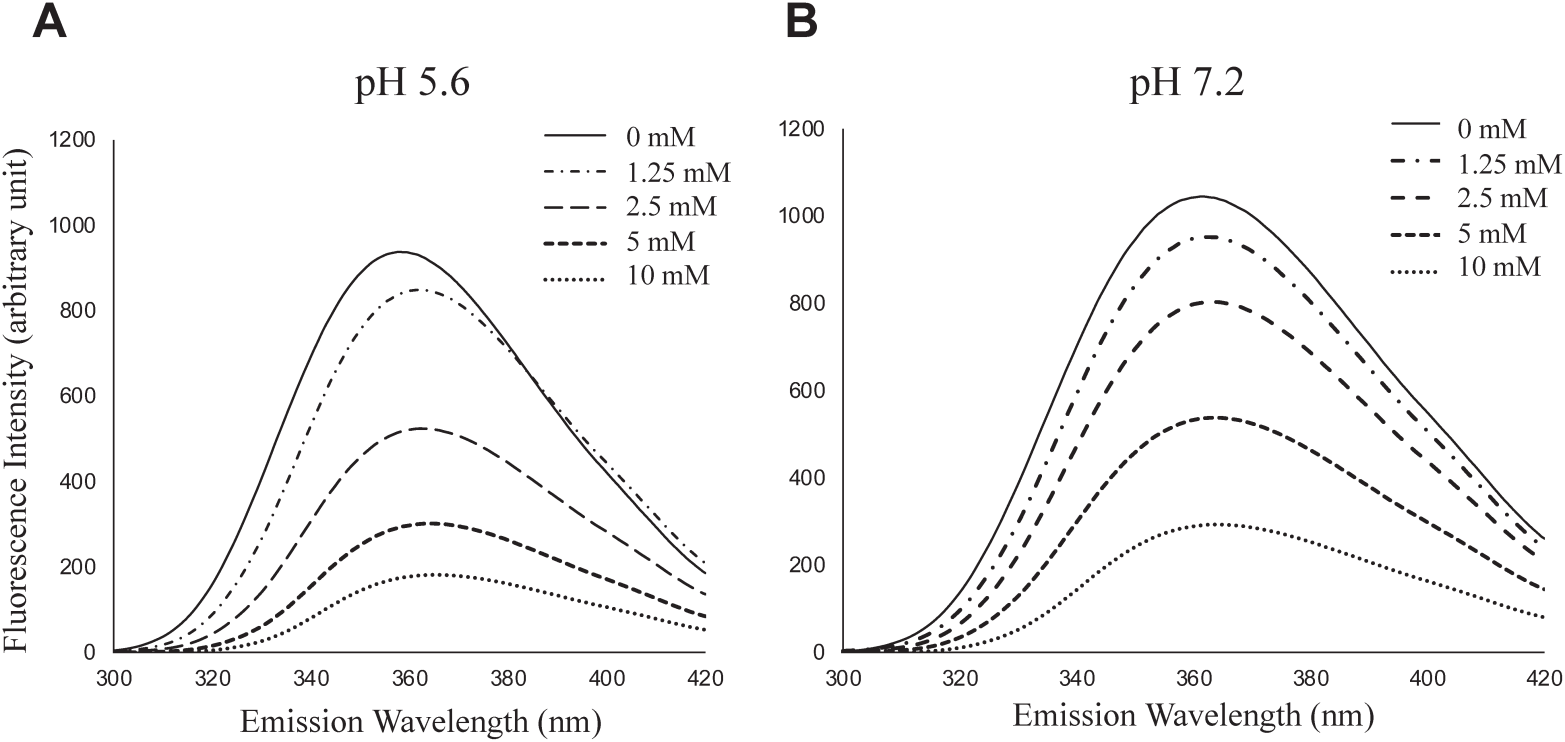
Figure 2. Changes in the fluorescence spectra of IAA upon addition of a plant complex-type free *N*-glycan, M3FX. A, Changes in the fluorescence spectra of IAA upon addition of M3FX (1.25, 2.5, 5.0, and 10 mM) at pH 5.6. B, Changes in the fluorescence spectra of IAA upon addition of M3FX at neutral pH.

In contrast, the addition of 10 mM sucrose (Figure 3A), lactose (Figure 3B), GlcNAc_2_ (Figure 3C), or GlcNAc_4_ (Figure 3D) at an acidic pH did not cause remarkable quenching of the IAA fluorescence, indicating that these oligosaccharides did not interact with or bind tightly to IAA. The specific interaction of plant complex-type *N*-glycans with IAA is thought to be due to the hydrophobic interactions between the hydrophobic part of the FNGs and the indole ring. The acetal rings of sugar residues have both hydrophilic and hydrophobic surfaces (Yano et al. 1988). Hydrophobic interactions between *N*-glycans linked to erythropoietin (EPO) and hydrophobic regions of EPO molecules have been reported (Toyoda et al. 2002). Although the spread of the hydrophobic surfaces formed on the FNG structure seems to be necessary for its interaction with IAA, the interaction between FNG and IAA cannot be explained by hydrophobicity alone, as some chitooligosaccharides, which are more hydrophobic than *N*-glycans, do not interact with IAA. The interaction of several plant complex FNGs with IAA was analyzed to confirm whether Xyl and/or Fuc residues are required for this interaction. Considering the quantitative limitations of these synthetic FNG samples and the cytosolic function of auxin, the analysis was performed at a neutral pH using a 3 mM concentration. As shown in Figure 4, GNM3FX (A), GNM3X (B), GNM3F (C), and GNM3 (D) caused the fluorescence quenching of IAA at a concentration of 3 mM at neutral pH, which was comparable to that of M3FX (2.5 mM). These results suggest that the characteristic common structure, the trimannosyl core structure bearing the GlcNAc_2_ unit, is necessary for the interaction of FNGs with IAA, but the occurrence of Xyl and/or Fuc residues is not. Because GlcNAc_2_ did not interact with IAA, the reducing terminal portion of GN2-FNGs does not appear to be directly involved in the interaction with IAA. The trimannosyl core sugar chain (Man_3_GlcNAc_2_, M3) is rarely found as a free sugar chain in plants and was not available at the time of this study, so interaction analysis with IAA could not be performed for this sugar chain. However, GNM3FX (Figure 4A) and GNM3 (Figure 4D) interacted with IAA as well as M3FX, suggesting that the non-reducing terminal GlcNAc residue was not involved in the interaction with IAA. Therefore, it seems reasonable to assume that M3 also interacts with IAA. In addition to M3, whether Man_1_GlcNAc_2_ or Man_2_GlcNAc_2_ (rarely found as FNGs in plants) bind IAA could not be analyzed in this study because not enough samples were available. However, the linear structure of Man_1-2_GlcNAc_2_ is similar to that of GlcNAc_4_, so it is unlikely that it binds tightly to IAA.

**Figure 3:**
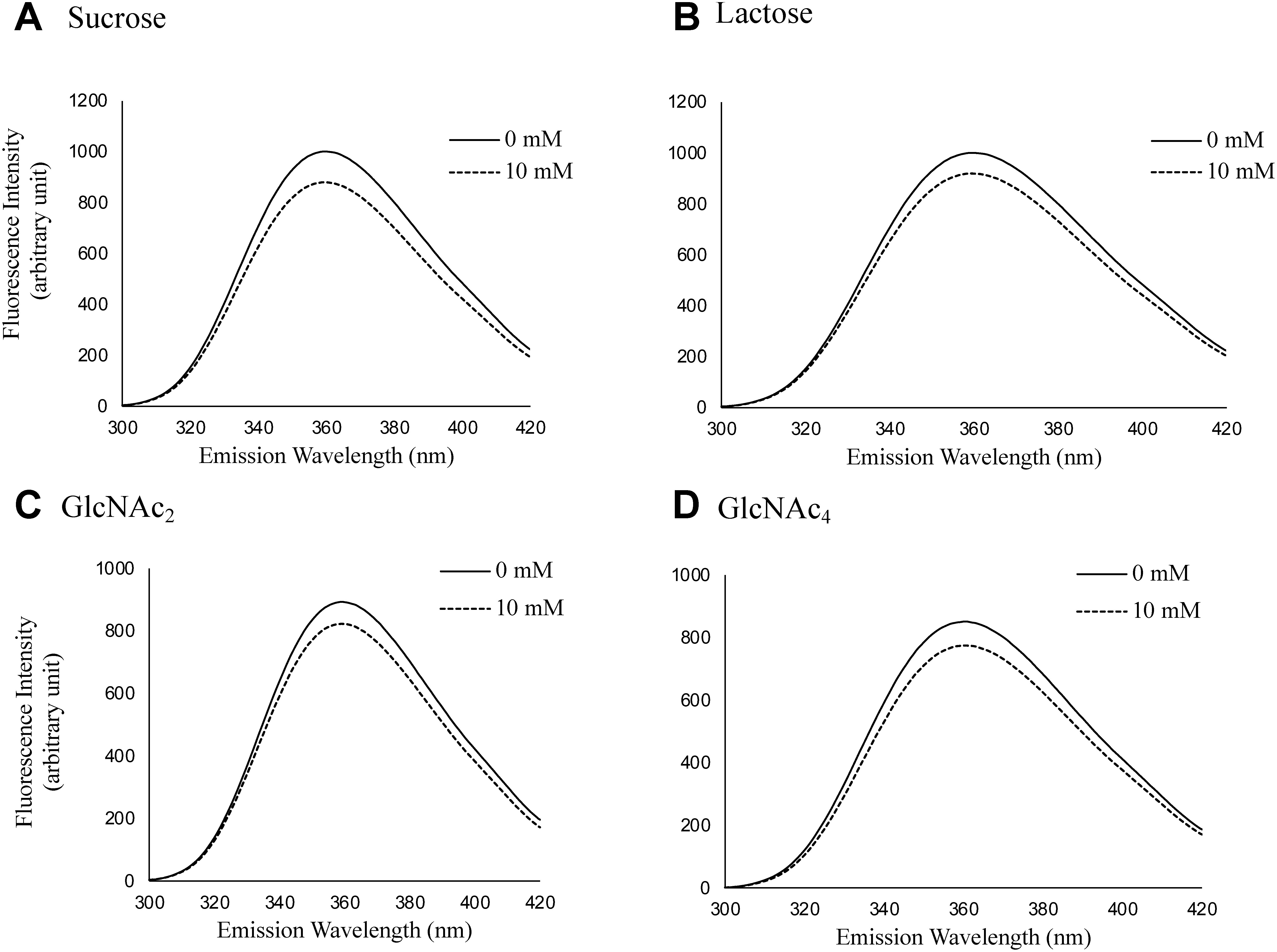
Figure 3. Fluorescence spectra of IAA in the presence of sucrose, lactose, and some *N*-acetyl chito-oligosaccharides (GlcNAc_2_, GlcNAc_4_). Each saccharide was added to a final concentration of 10 mM at pH 5.6. A, sucrose; B, lactose; C, GlcNAc_2_; D, chitotetraose (GlcNAc_4_).

**Figure 4:**
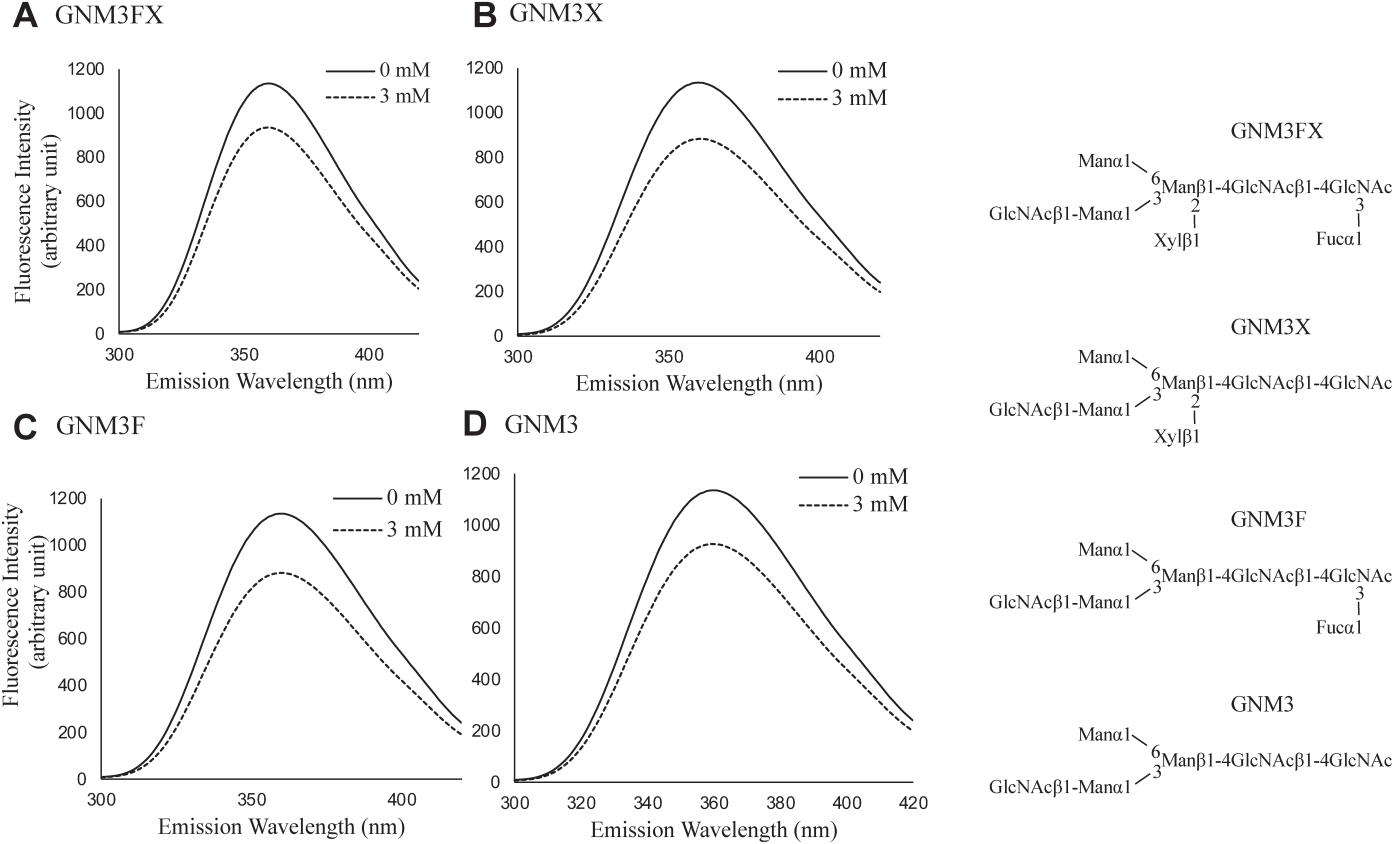
Figure 4. Changes in the fluorescence spectra of IAA upon addition of several plant complex-type free *N*-glycans at neutral pH. A, GNM3FX; B, GNM3X; C, GNM3F; D, GNM3.

In contrast to the plant complex-type free *N*-glycans, it has been reported that GN1-HMT-FNGs, which are produced by a combination of cytosolic PNGase and ENGase from misfolded glycoproteins (Fischl et al. 2011; Kimura et al. 2011; Maeda et al. 2021; Okamoto et al. 2022; Shirai et al. 2021; Suzuki and Funakoshi 2006), occur in the cytosol at relatively high concentrations (Kimura et al. 2011). Therefore, we sought to determine whether HMT-FNGs interacted with IAA, which functions as an important signaling molecule in the cytosol. As shown in Figure 5, we found that both GN1-HMT FNG (Man_9_GlcNAc_2_) and GN2-HMT FNG (Man_9_GlcNAc_1_), as well as M3FX, interacted with IAA at pH 7.2 (approximately cytosolic pH). Interestingly, the decrease in fluorescence intensity was slightly larger for the GN2 form than the GN1 form, suggesting that the number of GlcNAc residues at the reducing end may also affect this interaction. Although it is believed that the indole ring of tryptophan residue in the substrate binding site of lectins or glycosidases forms the hydrophobic interaction with a hydrophobic side of the substrate sugar residues (Nelson and Cox 2013), the modes of interaction between IAA and various FNGs may be clarified in detail by nuclear magnetic resonance and isothermal titration calorimetry using various GN1-FNGs and GN2-FNGs in future studies.

**Figure 5:**
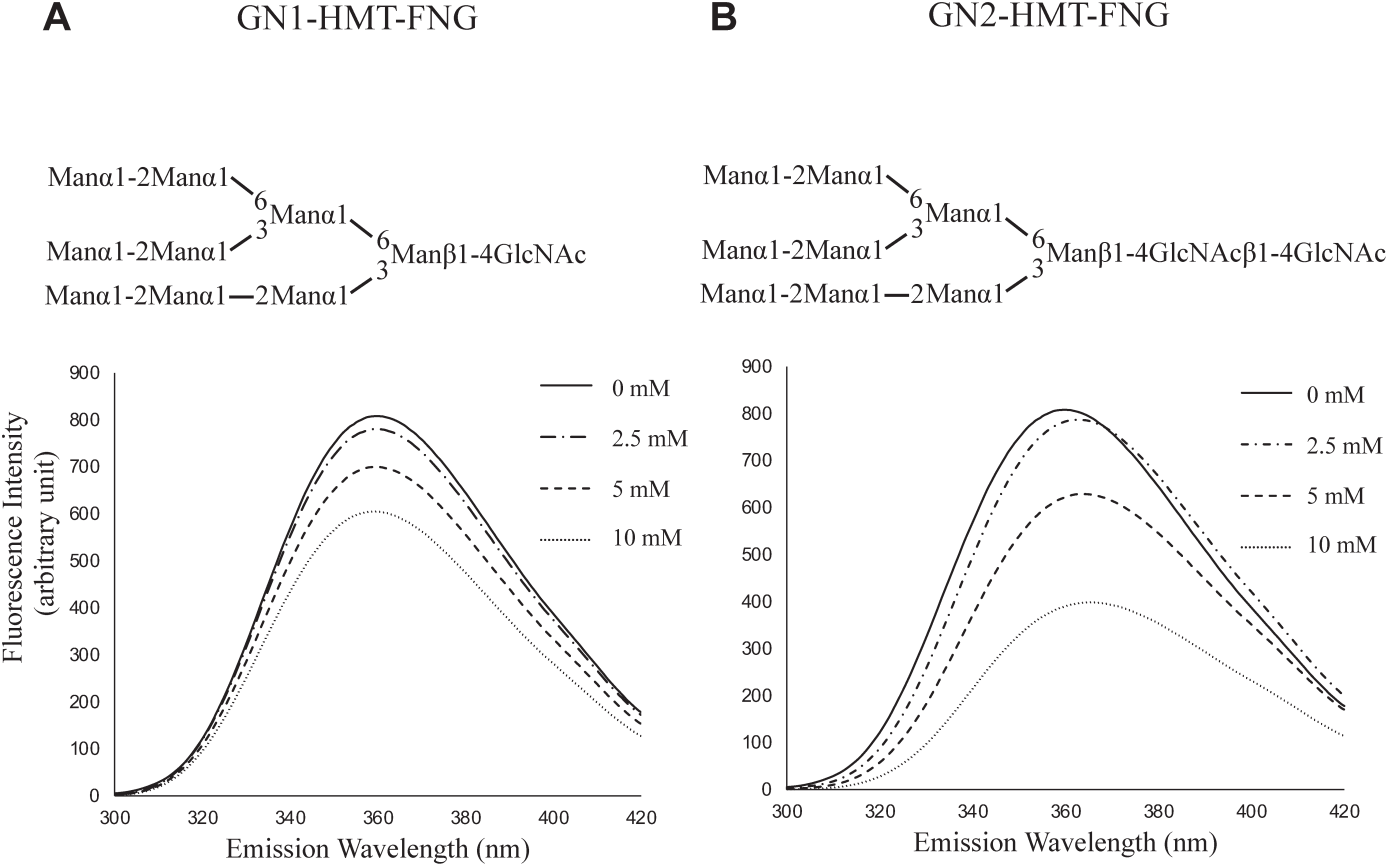
Figure 5. Changes in the fluorescence spectra of IAA upon addition of the high-mannose-type free *N*-glycan (HMT-FNG). A, Changes in the fluorescence spectra of IAA upon the addition of GN1-HMT-FNG (1.25, 2.5, 5.0, and 10 mM). B, Changes in the fluorescence spectra of IAA upon the addition of GN2-HMT-FNG.

The physiological significance of the interactions between IAA and FNGs remains unclear. Given that the intercellular movement of IAA is mediated by the polar transport system, the auxin is taken up into the cytosol (a neutral environment) by AUX1 (Yang et al. 2006), a channel in the cell membrane, and expelled in one direction into the extracellular space (an acidic environment) by PIN (Petrásek et al. 2006). During this intercellular trafficking, auxin is exposed to the acidic conditions (pH 5.0–5.5; pH 3.0–4.0 in the case of fruit) of the extracellular fluid (Grignon and Sentenac 1991). Because IAA with a p*K_a_* of 4.8 is assumed to be hydrophobic in the acidic extracellular space or fruits, binding to the hydrophilic FNGs is expected to confer hydrophilicity to IAA or reduce the hydrophobic property of IAA in the acidic environment as an IAA-FNG complex and facilitates the movement of IAA-FNGs complex in the aqueous environment under acidic conditions. Although the dissociation mechanism of IAA from PCT-FNG near the AUX1 transporter is obscure, if the pH near the transporter is slightly higher than in other spaces in the apoplast, the hydrophobic interaction between PCT-FNG and IAA may become slightly weaker, as shown in Figure 2. Regarding the uptake of IAA by the AUX1 transporter, it is possible that the specific interaction between IAA and the transporter is stronger than the nonspecific hydrophobic interaction between IAA and PCT-FNG, and that the FNG seems not to prevent IAA uptake into the cytosol.

A characteristic structural feature of plant complex-type *N*-glycans is the presence of β1-2Xyl and α1-3Fuc residues. The M3FX structure is more resistant to plant α-mannosidase than HMT-FNGs because of the presence of β1-2Xyl/α1-3Fuc residues. The major FNG present in the tomato xylem sap, which contains several acidic exoglycosidases (α-fucosidase, α-mannosidase, β-*N*-acetylglucosaminidase, and β-galactosidase), is M3FX (Tsujimori et al. 2019), and thus, the metabolic degradation of M3FX seemed to be slow. The importance of the β1-2Xyl residue in plant complex-type *N*-glycans for the growth of rice plants under abiotic stress conditions has been reported. Mutant β1-2xylosyltransferase-deficient rice plants exhibit dwarfism (Takano et al. 2015). The presence of β1-2Xyl/α1-3Fuc residues may delay the rate of M3FX degradation in the extracellular space. Trisaccharide and tetrasaccharide structures, such as Man_1_GlcNAc_2_ and Man_2_GlcNAc_2_, which are degradation intermediates without β1-2Xyl/α1-3Fuc residues and are easily degraded to monosaccharides, have rarely been found in plant cells.

Recently, several membrane-bound receptors for extracellular auxin have been identified and these extracellular receptors are assumed to play a critical role in the auxin-mediated signal transduction involved in plant growth (Yu et al. 2023). Therefore, it is also possible that the IAA-M3FX complex may be involved in IAA signaling by acting as a ligand for the IAA-extracellular receptor, although the detail mechanism of the IAA-signaling associated with the extracellular receptors remains to be elucidated.

The interaction of HMT-FNGs with IAA led us to the speculation that the HTM-FNGs may be involved in the physiological function of IAA in the cytosol. Since the HMT-FNGs occur in the cytosol at concentrations ranging from a few hundred nanomolar (nM) to a few micromolar (μM) (Kimura and Matsuo 2000; Kimura et al. 1997, 2002; Maeda et al. 2010), IAA incorporated into the cytoplasm is expected to bind readily to FNG. IAA has been shown to act as an important signaling molecule by binding to an F-box protein, TIR11 (Dharmasiri et al. 2005; Kepinski and Leyser 2005), in the cytosol and stimulates the degradation of the AUX/IAA repressor by the proteasome. In the interaction of TIR1 and the repressor protein, IAA is known to play an essential role as a glue. Therefore, it is feasible that the IAA-HMT-FNG complex may have some effect on the binding of TIR1 and the AUX/IAA repressor. Crystallographic analysis of the TIR1-IAA-FNG-substrate peptide (Aux/IAA) (if crystals can be formed) will provide new insights into the function of the interaction of IAA and HMT-FNGs in the plant cytosol.

In this study, we found that the plant hormone IAA interacts with FNGs that occur ubiquitously in developing plants. However, it is unclear whether *N*-glycans linked to proteins (not free *N*-glycans) interact with IAA. To the best of our knowledge, no study has analyzed the interaction of IAA with *N*-glycans linked to proteins. Since the core structure of the bound *N*-glycan is surrounded by a polypeptide or protein moiety, the spatial flexibility of the *N*-glycan is more restricted than that of the free sugar chain and interaction with IAA is less likely to occur.

## Abbreviations

Con A: concanavalin A
Endo-H: endo-β-*N*-acetylglucosaminidase from *Streptomyces plicatus*
ENGase: endo-β-*N*-acetylglucosaminidase
FNG: free *N*-glycan
Fuc: L-fucose
GlcNAc: *N*-acetyl-D-gluosamine
GN2-FNG: free *N*-glycan bearing *N,N*′-diacetylchitobiosyl unit at the reducing end
GN1-FNG: free *N*-glycan bearing one GlcNAc residue at the reducing end
GNM3: Manα1-6(GlcNAcβ1-2Manα1-3)Manβ1-4GlcNAcβ1-4GlcNAc
GNM3F: Manα1-6(GlcNAcβ1-2Manα1-3)Manβ1-4GlcNAcβ1-4(Fucα1-3)GlcNAc
GNM3FX: Manα1-6(GlcNAcβ1-2Manα1-3)(Xylβ1-2)Manβ1-4GlcNAcβ1-4(Fucα1-3)GlcNAc
GNM3X: Manα1-6(GlcNAcβ1-2Manα1-3)(Xylβ1-2)Manβ1-4GlcNAcβ1-4GlcNAc
HMT-FNG: high-mannose type FNG
IAA: indole-3-acetic acid
Man: D-mannose
M3FX: Manα1-6(Manα1-3)(Xylβ1-2)Manβ1-4GlcNAcβ1-4(Fucα1-3)GlcNAc
Man_3_GlcNAc_2_ (M3): Manα1-6(Manα1-3)Manβ1-4GlcNAcβ1-4GlcNAc
Man_9_GlcNAc_2_ (M9): Manα1-2Manα1-6(Manα1-2Manα1-3)Manα1-6(Manα1-2Manα1-2Manα1-3)Manβ1-4GlcNAcβ1-4GlcNAc
PCT-FNG: plant complex type FNG
PNGase: peptide:*N*-glycanase
Xyl: D-xylose
